# Effects of pre-partum dietary crude protein level on colostrum fat globule membrane proteins and the performance of Hu ewes and their offspring

**DOI:** 10.3389/fvets.2022.1046214

**Published:** 2022-11-30

**Authors:** Zhongyu Wang, Nana Zhang, Fadi Li, Xiangpeng Yue

**Affiliations:** State Key Laboratory of Herbage Improvement and Grassland Agro-Ecosystems, Key Laboratory of Grassland Livestock Industry Innovation, Ministry of Agriculture and Rural Affairs, Engineering Research Center of Grassland Industry, Ministry of Education, College of Pastoral Agriculture Science and Technology, Lanzhou University, Lanzhou, China

**Keywords:** growth performance, Hu sheep, colostrum, iTRAQ, MFGM proteins

## Abstract

Dietary proteins play important roles in the growth and reproduction of sheep, and the ewe's demand for proteins increases dramatically during late pregnancy. This research aimed to investigate the effect of dietary crude protein (CP) levels during late pregnancy on colostrum fat globule membrane (MFGM) protein and the growth performance of Hu sheep and their offspring, and provide a reference for the protein intake of ewes during late pregnancy. A total of 108 multiparous Hu sheep (45.6 ± 1.18 kg) were selected for this study, then 60 pregnant ewes confirmed by B-scan ultrasonography were randomly divided into three treatments (20 ewes/treatment) and fed by total mixed ration pellet with CP levels at 9.00% (LP), 12.0% (MP), and 15.0% (HP) during late pregnancy, respectively. The weight and dry matter intake of ewes during late pregnancy were recorded to calculate the average daily gain (ADG) and feed conversion ratio (FCR). Twin lambs were weighed on days 0, 7, 14, 30, 60, and 180 after birth to calculate ADG. Meanwhile, the colostrum of ewes was collected within 12 h after delivery. The colostrum MFGM proteins were identified and quantified by the isobaric tag for relative and absolute quantification (iTRAQ) coupled with liquid chromatography-tandem mass spectrometry methods. In addition, biological functions of differentially expressed proteins (DEPs) were annotated by Gene Ontology annotation and Kyoto Encyclopedia of Genes and Genomes pathway enrichment analysis. The results revealed that a 15.0% CP level had significant effects on the BW of lambs on days 0, 7, and 30 (*P* < 0.05). Notably, a total of 1,529 MFGM proteins were identified and 286 DEPs were found among three treatments. Functional analysis showed that DEPs were mainly involved in cell growth, differentiation, and tissue repair, and involved in metabolic pathways, such as the porphyrin and chlorophyll metabolism pathways. In this study, lambs in HP treatment had better growth performance; moreover, dietary 15.0% CP level also affected the colostrum MFGM proteins composition of Hu ewes. These observations can facilitate future studies on the feeding regimen of ewes during late pregnancy.

## Introduction

The nutrient requirements, especially protein requirements for ewes increase dramatically during the last 2 months of pregnancy (the late gestation) because this stage achieves 80% of the fetal growth and the development of the mammary gland for colostrum production (1). Previous studies showed that the appropriate protein nutrition for ewes during pregnancy has a positive effect on ewes' postpartum weight, colostrum production (2), and lambs' birth weight (3), and thus contributes to neonatal growth and development (4). Therefore, it is important to study the effect of ewes' protein intake during late pregnancy on milk composition and offspring growth.

As the main nutritional source for lambs at the early stage (5), the nutritive value of breast milk, particularly colostrum, is very important for lamb survival and growth. As an important component of colostrum, the composition and content of milk protein are crucial for newborn lambs. Milk fat globule membrane (MFGM) proteins are the components of milk fat globule membrane that are encapsulated on the surface of milk fat droplets, accounting for 1–2% of total milk protein (6). Despite the low content, MFGM proteins have a wide range of bio-functional properties, such as cell signal transduction, fat and protein transport, metabolic regulation, and other biological processes (7, 8). MFGM proteins also play important roles in modulating cell activity, promoting cell growth, and the defense functions of human, bovine, and goat newborns (9, 10).

Previous studies on comparative proteomics of MFGM proteins mostly focused on human and bovine milk (11, 12), and major functional proteins in the MFGM from human and bovine milk have already been extensively characterized and reviewed (7, 13). These functional proteins, such as butyrophilin, xanthine dehydrogenase/oxidase, and mucin, are combined with, embedded in, or attached to the MFGM (6, 14). Mucin 1, lactophorin C-terminal fragment, and PAS-6/7 can be used as antibiotics and anticancer agents, preventing physical damage and harmful microbial invasion (15). Since goat milk is one of the main milk sources for human consumption worldwide (16), several studies have also investigated the differences in MFGM proteomes between goats and other species (17, 18) and goats in different lactation periods (10). Previous studies on the characterization of MFGM proteins in goat colostrum and mature milk (10) have found that colostrum contains high levels of acute phase reactive proteins, such as complement component 3, lipopolysaccharide binding protein, and fibronectin 1, which contribute to the establishment of an anti-infective immune system in newborns. However, the MFGM proteins in sheep milk have been subjected to significantly less intense research effort, especially the effects of different dietary crude protein (CP) levels on the MFGM proteomes.

Hu sheep is one of the most widely distributed indigenous sheep breeds in China, which is well-known for sexual precocity, good lactation performance, and high fecundity (19). However, the nutritional requirements of Hu sheep during late pregnancy have not been well-established in China, which limits the development of feeding programs for the ewes during late pregnancy under housing conditions (3). Therefore, the current study was conducted to investigate the effects of dietary CP levels during late pregnancy on the growth performance and colostrum MFGM of Hu ewes and their offspring, and thus to maximize their production potential and economic benefits.

## Materials and methods

### Ethics statement

The animal procedures in this study were performed according to the Guidelines for the Care and Use of Laboratory Animals in China. This study was approved by the Animal Ethical and Welfare Committee of Ruminant Research Institution (Lanzhou University, approval number: CY20221001). All efforts were taken to minimize animal suffering.

### Animal management and dietary treatments

A total of 108 Hu sheep with similar ages (2.0 ± 0.3 yr) and body weight (BW) (45.6 ± 1.18 kg) were provided by Pukang Agriculture Ltd (Wuwei City, Gansu Province, China). Estrus and artificial insemination were synchronized to ensure a consistent pregnancy date. The day of insemination was considered day 0 of pregnancy. On pregnancy day 90, 60 pregnant ewes confirmed by B-scan ultrasonography were allocated to three dietary treatments (20 ewes/treatment) and were fed by total mixed ration (TMR) pellet with CP levels of 9.00% (low protein treatment, LP), 12.0% (medium protein treatment, MP), and 15.0% (high protein treatment, HP), respectively. The selection of the three protein levels was referred as described previously (20) with slight modifications. The ingredients and nutrient compositions of the diets are shown in Table 1, the soybean meal (CP: 47%) was the main CP source. The N content in the diets was analyzed using a protein analyzer (K9840, Hanon Advanced Technology Group Co., Ltd, Jinan, China) according to the Kjeldahl method and crude protein was calculated as N × 6.25. Neutral detergent fiber and acid detergent fiber were determined according to the method of Van Soest et al. (21). The calcium content was determined by atomic absorption spectrophotometry. The phosphorus content was measured according to the method of Fiske and Subbarow (22). The formal experiment was started 14 days after adaptation to the experimental diets. Thus, the formal experimental period was started at 105 d.

**Table 1:** Ingredients and nutrient levels of the experimental diets with three different protein levels (dry matter basis).

**Item**	**Diet^a^**		
	**LP**	**MP**	**HP**
**Ingredients, %**			
Sunflower hull	8.00	18.0	29.0
Corn straw	36.0	26.0	15.0
Corn germ cake	20.0	11.0	3.00
Corn grain	30.0	30.0	30.0
Soybean meal	2.40	8.00	10.0
Corn gluten meal	0.00	3.50	9.40
Limestone	0.20	0.20	0.20
Calcium bicarbonate	0.30	0.20	0.30
Salt	0.60	0.60	0.60
Premix^b^	2.50	2.50	2.50
Total	100	100	100
**Nutrient levels^c^**			
Crude protein (%)	9.00	12.0	15.0
Neutral detergent fiber (%)	41.3	39.7	38.4
Acid detergent fiber (%)	22.9	23.9	24.6
Metabolizable energy^d^ (MJ.kg^−1^)	10.3	10.3	10.3
Calcium (%)	0.79	0.79	0.79
Phosphorus (%)	0.28	0.29	0.28

a LP, low protein; MP, medium protein; HP, high protein.

b Premix provided per kg of feed: Vitamin A, 2500 IU; Vitamin E, 23 IU; Cu, 69.63 mg; Fe, 69.63 mg; Zn, 55 mg; Mn, 23.70 mg; I, 0.67 mg; Co, 0.3 mg; and Se, 0.3 mg.

c Except for metabolizable energy was calculated value, the rest were measured values.

d Metabolizable energy = total digestible nutrients × 0.04409 × 0.82, according to the National Research Council (23).

All of the experimental ewes were housed in clean pens, and water was provided freely during the entire experimental period. Rations of the same weight were provided three times a day at 8:00, 14:30, and 20:00 for *ad libitum* intake and 10% refusals on an as-fed basis. After birth, newborn lambs were left to suckle their dams freely for the first 3 days and were raised with ewes till weaning at 60 days of age, and then fed by the same commercial pellet lamb feed (protein level 19%) from d 60 to 90, and commercial pellet fatten feed (protein level 14.5%) from d 90 to 180. As the average litter size of Hu sheep is 2.06 (19), ewes giving single lambs and triple lambs and above were rare in our experiment. Twin lambs and their ewes were chosen for subsequent analysis, which left 12 ewes in LP, 10 in MP, and 15 in HP treatments.

### Growth performance

The ewes were weighed on days 105 and d 140 before feeding in the morning. Dry matter intake (DMI) was recorded daily by weighting feed offered and refused for ewes. Growth performance was evaluated by calculating the average daily gain (ADG) and feed conversion ratio (FCR). The formulas for calculation were as follows: ADG (g/d) = (final BW – initial BW)/days; FCR = DMI (g/d)/ADG (g/d). Twin lambs were weighed on days 0, 7, 14, 30, 60, and 180 after birth to calculate ADG.

### Sample collection and preparation of the MFGM proteins

The colostrum (15 mL/ewe) of ewes with a litter size of two mentioned above was collected by manually milking within 12 h after delivery. To reduce the individual difference, the colostrum samples from each treatment were mixed (18). Then, the colostrum was stored at −20°C until the MFGM protein analysis.

The extraction of MFGM proteins from colostrum was performed as described previously (18) with slight modifications. Two colostrum samples (4 mL/sample) were taken from each treatment for the extraction. Briefly, 4 mL of colostrum samples were centrifuged at 4,000 × g for 30 min at 4°C to separate the milk fat. The upper layer was transferred to a new tube and washed with 100 mM, pH 7.4 phosphate-buffered saline solution (1:5, v/v) under sonication. The mixture was then centrifuged at 4,000 × g for 30 min at 4°C and the supernatant was collected. The 0.4% sodium dodecyl sulfate (1:1, v/v) was added to the supernatant and was sonicated for 1 min. The mixture was centrifuged at 10,000 × g for 10 min at 4°C to obtain the MFGM proteins. The concentration of MFGM proteins was determined by using a bicinchoninic acid protein assay kit (PC0020, Solarbio Ltd., Beijing) according to the manufacturer's instructions.

### Protein digestion and isobaric tags for relative and absolute quantitation labeling

The MFGM proteins were digested by filter-aided sample preparation (24). Briefly, 30 μL of MFGM proteins from each sample were reduced with DTT at a final concentration of 100 mM and boiled in a water bath for 5 min. The samples were mixed with Urea buffer solution (200 μL, 8 M Urea, and 150 mM Tris-HCl, pH 8.0) after cooling to room temperature, loaded on an ultra-centrifugal filter (Pall units, 10 kD), and centrifuged at 14,000 × g for 15 min. This step was repeated once. Then, the samples were mixed with 100 μL of iodoacetamide solution (100 mM iodoacetamide in UA buffer) and shocked at 600 rpm for 1 min, held at room temperature in the dark for 30 min, and centrifuged at 14,000× g for 15 min. A 100 μL aliquot of dissolution buffer (50 mM triethylammonium bicarbonate, pH 8.5) was used to wash the filters by centrifugation at 14,000× g for 15 min. This wash step was repeated twice. After adding 40 μL of 10% trypsin buffer (Promega, Southampton, UK), the protein suspensions were shocked at 600 rpm for 1 min and digested at 37°C for 16–18 h. The digestions were centrifuged at 14,000 × g for 15 min to collect the resulting peptides in the filtrate.

The peptides were labeled with eight-channel iTRAQ reagent (AB SCIEX, Massachusetts, USA) according to the manufacturer's instructions. The protein samples were labeled as (Sample-L1)-113, (Sample-L2)-114, (Sample -M1)-115, (Sample -M2)-116, (Sample -H1)-117, and (Sample -H2)-118, and the labeling solution reaction was incubated at room temperature for 60 min.

### Peptide fractionation by strong cation exchange chromatography

All labeled peptides were mixed and fractionated using strong cation exchange chromatography and the AKTA Purifier 100 system (GE Healthcare, Milwaukee, WI, USA). The column was a 4.6 × 100 mm polysulfethyl column (5 μm, 200 Å) (PolyLCInc., Columbia, MD, USA), and buffer A (10 mM KH_2_PO_4_ pH 3.0 in 25% CAN), buffer B (500 mM KCl and 10 mM KH_2_PO_4_ pH 3.0 in 25% CAN). The chromatographic column was equilibrated with buffer A, and the sample was applied to the chromatographic column. The flow rate was set to 1 mL/min. The solvents were applied using the time gradient from 0 to 100% buffer B and followed by 60 min at 0%.

The absorbance values were monitored at 214 nm during elution, and the fractions were collected every 1 min. The samples were freeze-dried and then desalted using C_18_Cartridges (66872-U, Sigma, St. Louis, MO, USA). All samples were stored at −80°C until the liquid chromatography (LC)–tandem mass spectrometry (MS/MS) analysis.

### Liquid chromatography–tandem mass spectrometry analysis using the Q exactive

The Easy nano-LC system (Thermo Scientific, Waltham, MA, USA) was used to separate the peptides. Dried peptides were re-suspended in mobile buffer A (0.1% formic acid). Then, 10 μL of the peptide mixture was injected and fractionated on a C18 analytical column (75 μm×100 nm, 3 μm) with a linear gradient elution of mobile buffer B (80% ACN containing 0.1% formic acid). The gradient was run at 300 nL/min from 0 to 45% of buffer B for 55 min, then changed up to 100% for 3 min, then maintained at 100% buffer B for 2 min.

Q-Exactive (Thermo Finnigan, San Jose, CA, USA) was used to analyze the separated peptides under the following conditions: polarity was positive and the default charge state was 2. Full MS was scanned from 300 to 1,800 mass/charge (m/z) with a resolving power of 70,000, and the maximum ion injection time for the survey scan was 50 ms. The resolution for data-dependent MS/MS spectra was set to 17,500 with a fixed first mass of 110 m/z. The normalized collision energy was 30 eV and the dynamic exclusion duration was 60 s.

### Database search and data analysis

Peptide identification from raw data was carried out using the MASCOT engine (Matrix Science, London, UK; version 2.2) and Proteome Discoverer software (Thermo Electron, San Jose, CA; version 1.4). The MS/MS spectra were searched against the UniProt sheep database (27,563 sequences, downloaded on 20/11/2021). The method for protein identification was referred as described previously (25). The following options were used: Peptide mass tolerance = 20 ppm, MS/MS tolerance = 0.1 Da, Enzyme = Trypsin, Missed cleavage = 2, Fixed modification: Carbamidomethyl (C), iTRAQ 8 plex (K), iTRAQ 8 plex (N-term), Variable modification: Oxidation (M) (25). Peptides were identified at a false discovery rate (FDR) ≤ 0.01. Differentially abundant proteins were determined using a Student's *t*-test. Proteins with *P* < 0.05 and a fold change ≥ 1.2 were considered upregulated, and a fold change ≤ 0.83 was considered to be downregulated.

### GO and KEGG analysis

Gene ontology (GO) functional annotations (http://geneontolog.org/) were performed using Blast2Go software (26). The differentially expressed MFGM proteins in the colostrum were classified into biological processes, molecular functions, and cellular components according to their GO annotations between paired comparisons of the three treatments. Kyoto encyclopedia of genes and genomes (KEGG) pathways analysis (https://www.genome.jp/kegg/) was performed using KAAS (KEGG Automatic Annotation Server) software (27). Functional enrichments were statistically analyzed using Fisher's exact test, and only *P*-value that was ≤ 0.05 were considered significantly enriched.

### Statistical analysis

Quantitative data obtained in the present study (except proteome analysis) were presented as the mean ± standard deviation and statistically analyzed using a one-way analysis of variance (ANOVA), and the significance of differences among means was determined using the Duncan multiple range test using SPSS 19.0 (SPSS Inc., Chicago, IL, USA). Statistical difference was considered significant at *P* < 0.05.

## Results

### Effect of dietary crude protein levels during late pregnancy on growth performance

The effects of dietary CP levels on the growth performance of Hu ewes during late pregnancy were shown in Table 2. The results revealed that protein levels had no effect (*P* > 0.05) on the final BW, DMI, ADG, and FCR of Hu ewes during late pregnancy.

**Table 2:** Effects of three different dietary protein levels on growth performance in Hu ewes during late pregnancy.

**Parameter^a^**	**Treatments**			***P*-value**
	**LP (*n* = 12)**	**MP (*n* = 10)**	**HP (*n* = 15)**	
105–140d				
Initial BW, kg	62.9 ± 1.71	58.9 ± 2.24	59.7 ± 1.53	0.28
Final BW, kg	71.5 ± 1.58	68.1 ± 2.11	70.6 ± 1.81	0.46
DMI, kg/d	2.50 ± 0.28	2.4 ± 0.33	2.32 ± 0.30	0.14
ADG, g/d	246 ± 2.45	264 ± 1.70	311 ± 2.37	0.11
FCR	8.62 ± 3.73	7.87 ± 1.79	7.76 ± 4.09	0.74

Values are expressed as means ± standard deviation.

a BW, body weight; DMI, dry matter intake; ADG, average daily gain; FCR, feed conversion ratio.

For the twin lambs, dietary CP levels had a significant effect on the birth weight (Table 3), indicating that higher protein level was positively associated with the birth weight of lambs (*P* < 0.05). The BW on d 7 and d 30 of lambs in the HP treatment was significantly greater than that in the LP treatment (*P* < 0.05).

**Table 3:** Effects of three different dietary protein levels on growth performance of twin lambs.

**Parameter^a^**	**Treatments**			***P*-value**
	**LP (*n* = 24)**	**MP (*n* = 20)**	**HP (*n* = 30)**	
0–7d				
Initial BW, kg	3.80 ± 0.11^b^	3.99 ± 0.11^b^	4.38 ± 0.13^a^	0.01
Final BW, kg	5.84 ± 0.14^b^	6.09 ± 0.16^ab^	6.42 ± 0.15 ^a^	0.03
ADG, g/d	292 ± 12.2	300 ± 11.5	292 ± 13.3	0.88
7–14d				
Initial BW, kg	5.84 ± 0.14^b^	6.09 ± 0.16^ab^	6.42 ± 0.15 ^a^	0.03
Final BW, kg	7.34 ± 0.16	7.60 ± 0.20	7.91 ± 0.19	0.12
ADG, g/d	214 ± 9.30	215 ± 12.75	214 ±8.00	0.99
14–30d				
Initial BW, kg	7.34 ± 0.16	7.60 ± 0.20	7.91 ± 0.19	0.12
Final BW, kg	10.2 ± 0.23 ^b^	10.3 ± 0.28^ab^	11.0 ± 0.26 ^a^	0.05
ADG, g/d	175 ± 6.76	171 ± 10.41	195 ± 9.54	0.16
30–60d				
Initial BW, kg	10.3 ± 0.23 ^b^	10.3 ± 0.28^ab^	11.0 ± 0.26 ^a^	0.05
Final BW, kg	17.5 ± 0.44	19.2 ± 0.75	19.3 ± 0.46	0.06
ADG, g/d	245 ± 10.62	297 ± 17.73	277 ± 11.68	0.06
60–180d				
Initial BW, kg	17.5 ± 0.44	19.3 ± 0.75	19.3 ± 0.46	0.06
Final BW, kg	38.6 ± 1.12	40.8 ± 1.59	43.3 ± 1.39	0.08
ADG, g/d	176 ± 7.43	180 ± 8.74	200 ± 9.76	0.14
0–180d				
Initial BW, kg	3.80 ± 0.11^b^	3.99 ± 0.11^b^	4.38 ± 0.13^a^	0.01
Final BW, kg	38.6 ± 1.12	40.8 ± 1.59	43.3 ± 1.39	0.08
ADG, g/d	193 ± 8.62	205 ± 8.62	217 ± 7.49	0.12

Values are expressed as means ± standard deviation.

a BW, body weight; ADG, average daily gain. Values in the same raw of the same item with different superscripts differ significantly (*P* < 0.05) and the same letter or no letter differs insignificant difference (P > 0.05).

### Effect of dietary protein levels during late pregnancy on MFGM proteins

**Figure 1:**
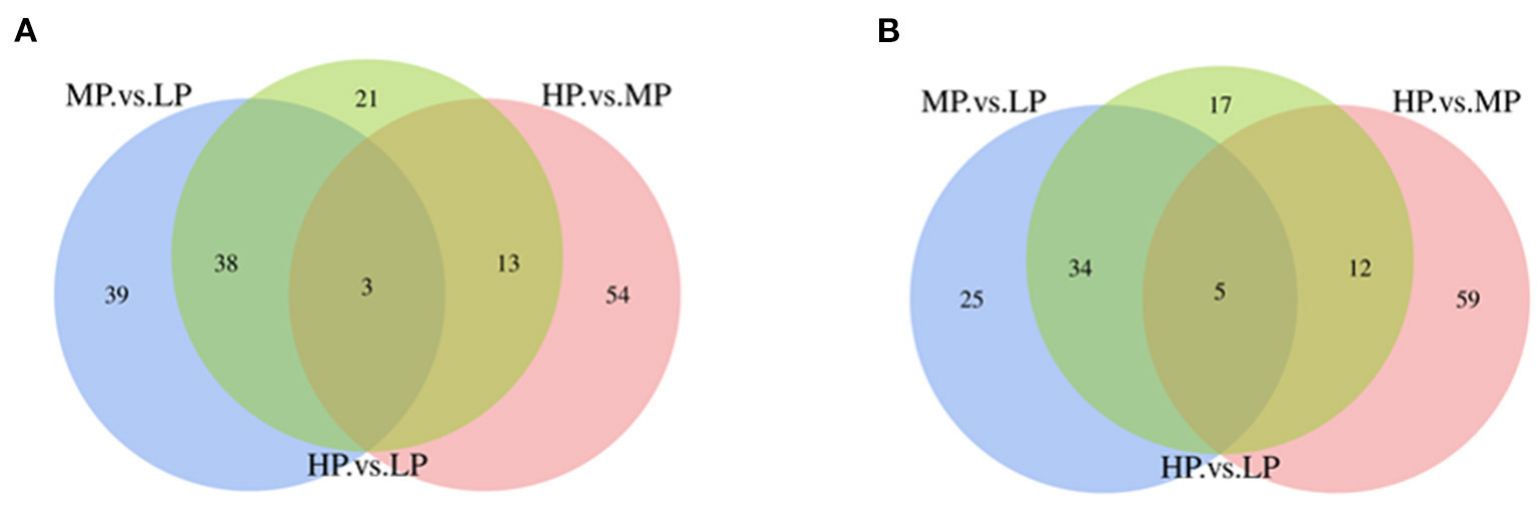
The Venn diagram of differentially expressed proteins. **(A)** The Venn diagram of differentially expressed up-regulated proteins; **(B)** Differentially expressed down-regulated protein Venn diagram.

Based on the iTRAQ LC-MS/MS method, a total of 173,772 secondary spectrograms were generated from 6 samples, which identified 5,949 peptides and 1,529 proteins in MFGM. Pair-wise comparisons among LP, MP, and HP treatments identified 286 differentially expressed MFGM proteins (DEPs, Supplementary Table 1), including 144 (80 up-regulated and 64 down-regulated) between MP and LP treatments, 146 (70 up-regulated and 76 down-regulated) between HP and MP treatments, and 143 (75 up-regulated and 68 down-regulated) between HP and LP treatments (Figure 1). Of those DEPs, 3 were consistently up-regulated, while 5 down-regulated with increasing dietary protein levels (Table 4), for instance, BPI1 domain-containing protein, EF-hand domain-containing protein, zinc finger FYVE-type containing 1, and dematin actin binding protein.

**Table 4:** The consistently up-regulated and down-regulated MFGM proteins with dietary protein level.

**No**.	**Accession no**.	**Protein name**	**Fold change^a^**			***P*** **-value**		
			**M/L**	**H/M**	**H/L**	**M/L**	**H/M**	**H/L**
1	W5P8F9	BPI1 domain-containing protein	1.81	1.48	2.68	0.04	0.01	< 0.01
2	W5QIW7	EF-hand domain-containing protein	4.73	7.89	37.25	< 0.01	< 0.01	< 0.01
3	W5NYU4	G_PROTEIN_RECEP_F1_2 domain-containing protein	2.43	1.48	3.59	< 0.01	< 0.01	0.01
1	W5NQ27	Zinc finger FYVE-type containing 1	0.43	0.63	0.27	< 0.01	0.01	< 0.01
2	W5QCI0	Low density lipoprotein receptor class A domain containing 3	0.50	0.68	0.34	< 0.01	0.04	< 0.01
3	W5PLJ2	Dematin actin binding protein	0.55	0.68	0.37	0.02	0.04	< 0.01
4	W5NTJ2	TPX2 microtubule nucleation factor	0.57	0.59	0.33	0.02	< 0.01	< 0.01
5	W5PD41	GIT ArfGAP 1	0.53	0.54	0.29	0.01	< 0.01	< 0.01

a Relative abundance of MFGM proteins in MP treatment vs. LP treatment, HP treatment vs. MP treatment, and HP treatment vs. LP treatment.

**Figure 2:**
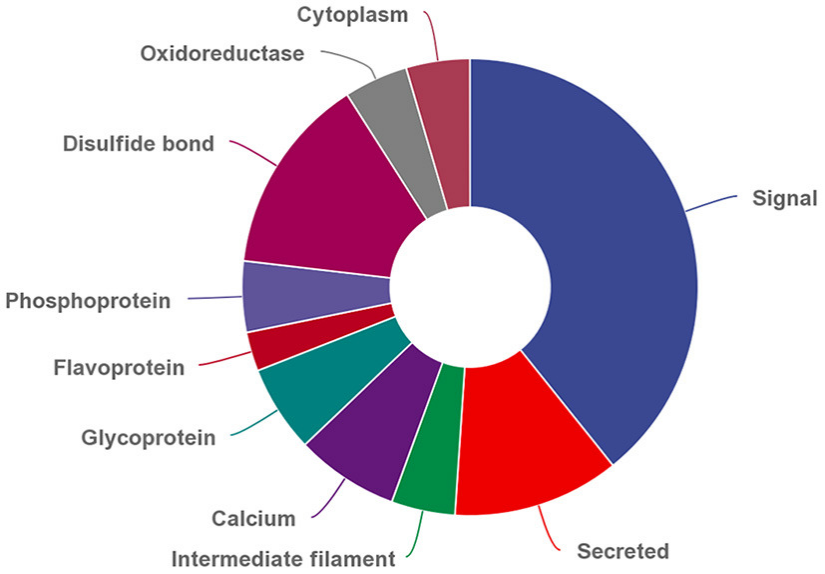
Top 10 functional categories of differential expressed MFGM proteins based on UP_KEYWORDS (https://david.ncifcrf.gov/home.jsp).

In addition, 286 DEPs can be classified into 25 functional categories based on their UP_KEYWORDS, and the top 10 functional categories were shown in Figure 2. The three most abundant differentially expressed MFGM proteins were signaling, disulfide-bonded, and secreted proteins with numbers of 70, 25, and 21, respectively.

### GO analysis of DEPs

**Figure 3:**
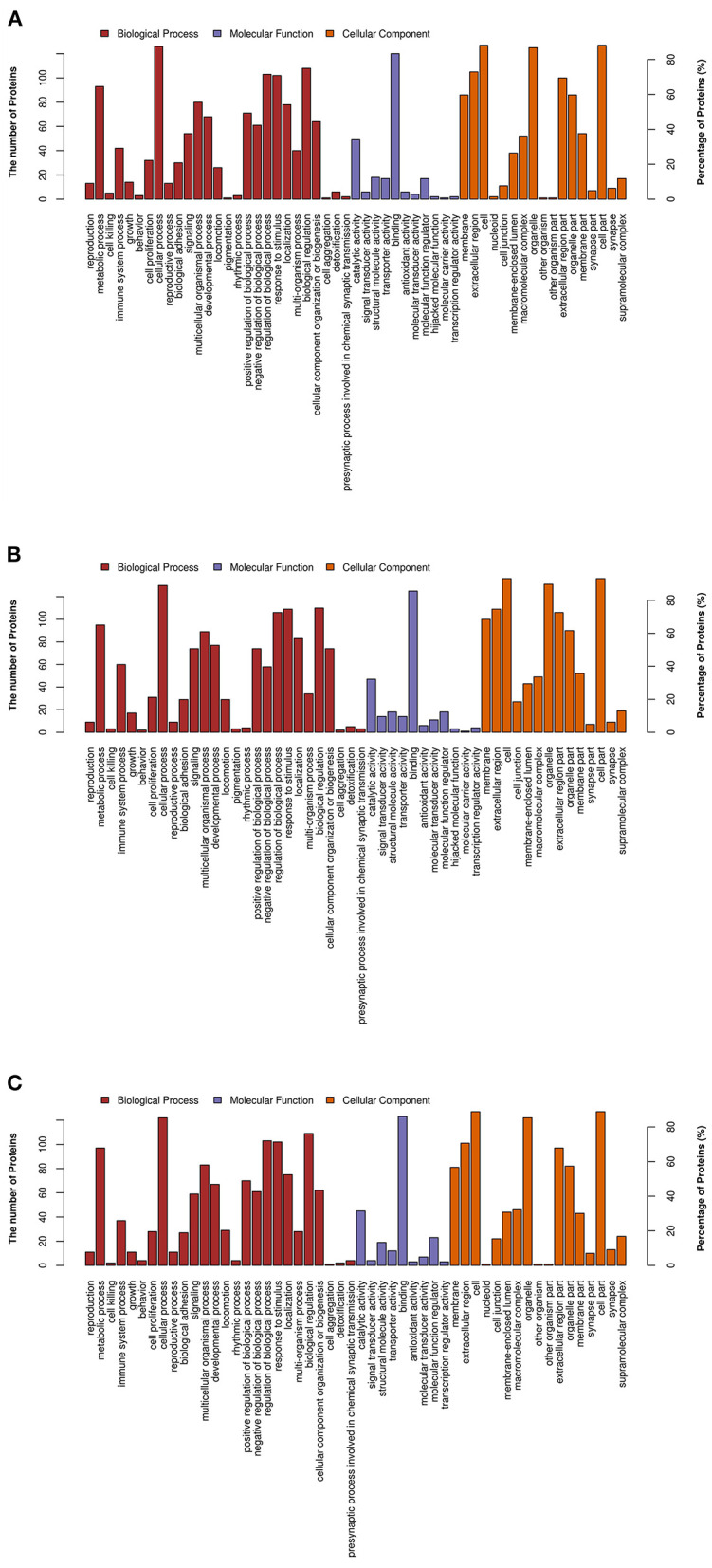
Gene ontology (GO) annotation of differentially expressed MFGM proteins. **(A)** The GO analysis of differential proteins in MP/LP treatment; **(B)** The GO analysis of differential proteins in HP/MP treatment; **(C)** The GO analysis of differential proteins in HP/LP treatment.

As shown in Figure 3, the DEPs identified between the MP and LP treatments, HP and MP treatments, HP and LP treatments were mostly enriched in similar GO pathways. The biological process subgroup, which contains the most MFGM proteins, mainly included cellular process, biological regulation, metabolic process, response to stimulus, and regulation of biological processes. The most common molecular function was binding and catalytic activity, followed by molecular function regulator, structural molecule activity, and transporter activity. Regarding cellular components, MFGM proteins mainly originated from the cell and organelles. In addition, macromolecular complex proteins, extracellular region proteins, and membrane-enclosed lumen proteins were identified in the colostrum MFGM proteins.

**Figure 4:**
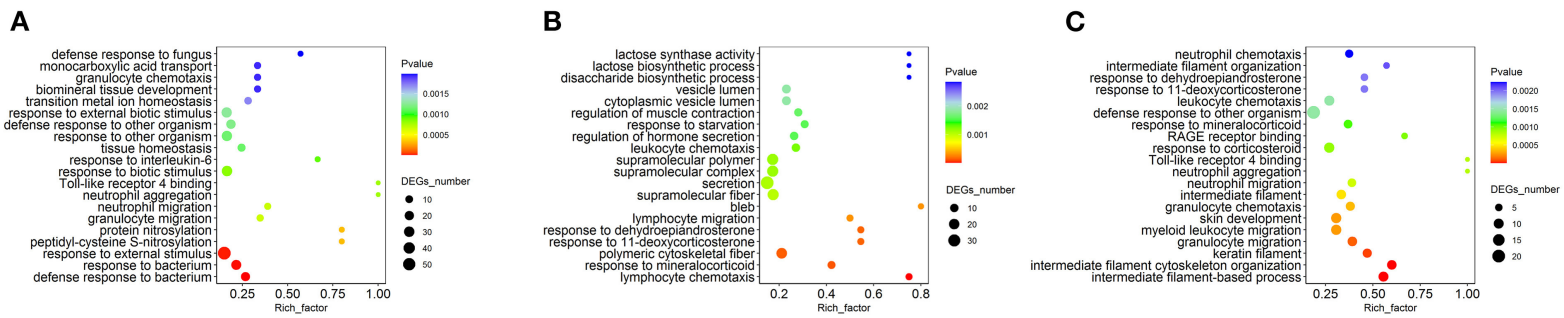
Gene ontology (GO) enrichment analysis of differentially expressed MFGM proteins. **(A)** The GO enrichment analysis of differential proteins in MP/LP treatment; **(B)** The GO enrichment analysis of differential proteins in HP/MP treatment; **(C)** The GO enrichment analysis of differential proteins in HP/LP treatment.

To further elucidate the functions of the differentially expressed MFGM proteins, the enrichment analysis based on the GO annotation system was performed (Figure 4). Compared with the other two intergroup comparisons, the DEPs identified between the HP and LP treatments were enriched in the functional term in lactose synthase activity, disaccharide biosynthetic process, and lactose biosynthetic process.

### KEGG pathway analysis of DEPs

**Figure 5:**
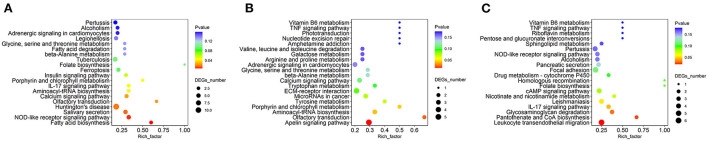
Kyoto Encyclopedia of Genes and Genomes (KEGG) pathway analysis of differentially expressed MFGM proteins. **(A)** The KEGG pathway analysis of differential proteins in MP/LP treatment; **(B)** The KEGG pathway analysis of differential proteins in HP/MP treatment; **(C)** The KEGG pathway analysis of differential proteins in HP/LP treatment.

As shown in Figure 5, the set of proteins was significantly enriched in nine pathways between the MP and LP treatments: Huntington's disease, the NOD-like receptor signaling pathway, salivary secretion, the calcium signaling pathway, fatty acid biosynthesis, porphyrin and chlorophyll metabolism, IL-17 signaling pathway, olfactory transduction, and aminoacyl-tRNA biosynthesis.

The set of proteins between the HP and MP treatments was significantly enriched in four pathways, including leukocyte transendothelial migration, pantothenate and CoA biosynthesis, the IL-17 signaling pathway, and glycosaminoglycan degradation.

The set of proteins between the HP and LP treatments was significantly enriched in four pathways, including the apelin signaling pathway, porphyrin, and chlorophyll metabolism, aminoacyl-tRNA biosynthesis, and olfactory transduction.

## Discussion

The nutrition of ewes in the pre-partum period not only affects the growth of the developing fetus, but also the capability of the ewe to supply the lamb with adequate amount of colostrum pre -partum (28). Therefore, the specific dietary changes of ewes were very important to the colostrum composition and subsequent physiological responses of growing lambs (3). Despite the revelations of previous studies, research on the characterization of sheep MFGM proteins is very limited, particularly studies on the effects of different nutritional levels on the MFGM proteome. This study evaluated the effects of pre-partum dietary CP levels on the weight of Hu ewes and their offspring and analyzed the differential expression of MFGM proteins in Hu ewes' colostrum at different dietary CP levels. The birth weight of the lambs in the HP treatment was significantly greater than that in the MP and LP treatments. In our study, 286 DEPs were identified, and all of them were fully annotated to illustrate the molecular functions of the sheep colostrum MFGM proteins during infant growth and development. This research enriches the sheep protein database and provides theoretical guidance for a feeding regimen for late pregnancy ewes under housing conditions.

Maternal nutritional levels during pregnancy may affect the growth and angiogenesis of the placenta, which, in turn, affects blood flow to the placenta and the absorption of nutrients by the fetus, ultimately affecting fetal growth and metabolism. Therefore, nutrition during pregnancy has an important effect on the growth and development of the fetus (29). The placenta is fully developed during late pregnancy. This time is a rapid growth period for the fetus. Maternal nutrition may affect the transport and distribution of fetal nutrient substrates, thus affecting birth weight and postnatal growth and development of the offspring (30). He et al. (31) reported that restricting protein reduces body weight gain in late pregnancy ewes (Liuyang black goat, local breed), and Ocak et al. (20) found a significant increase in body weight gain in ewes (Hampshire Down × Karayaka crossbred) fed 16.5% protein during late pregnancy. Thus, dietary protein levels during late pregnancy can affect the growth status of ewes. Similar conclusions were obtained in this study, where the ADG of ewes during late pregnancy increased and the FCR decreased with the enhancement of the dietary protein level. The results indicate that the ewes with a 15.0% dietary CP level had a high feed utilization rate, which can achieve better economic returns. Ocak et al. (20) reported that compared with the 11.7% CP level, the high CP level (16.5%) diets offered to the ewes during late pregnancy (day 85 of pregnancy to the end of delivery) increased lamb birth weight (4.3 ± 0.1 vs. 4.9 ± 0.1 kg; *P* < 0.05). Wang et al. (3) reported that compared with 10.12 and 11.26% CP levels, the high CP level diets (12.4%) offered to the Hu sheep during late pregnancy (day 90 of pregnancy to the end of delivery) increased lamb body size development (*P* < 0.05). This study also reached a similar conclusion, indicating that a 15.0% dietary CP level given to ewes during late pregnancy was more beneficial to fetal growth and increased the birth weight of lambs, which is inextricably linked with future weight gain and growth development.

Among the 286 differentially expressed MFGM proteins, a large number of proteins were related to post-translational modifications based on their UP_KEYWORDS, including phosphoprotein and glycoprotein. Phosphorylation and glycosylation are post-translational protein modifications, by which cells regulate gene expression, protein activity, and many physiological and pathological processes (32–34). In addition, many MFGM proteins accumulate in the cytoplasm, suggesting the source of the cellular components included in these MFGM proteins.

EF-hand domain-containing protein was one of the DEPs that we found persistently up-regulated with increasing CP levels, which is the most abundant calcium-binding protein (35). EF-hand domain-containing protein primarily plays a role in modulating the duration of calcium signaling and maintaining calcium homeostasis (36). Calcium regulates vital cellular functions, including gene expression, protein secretion, metabolism, and apoptosis in a wide variety of cells (36). Low density lipoprotein receptor class A domain containing 3 was one of the DEPs that we found persistently down-regulated with the increasing CP levels, which was associated with fat percentage and FA composition in Vrindavani cattle (37). Wang et al. (3) reported that the increase in dietary CP level during late pregnancy significantly increased the blood triglyceride of Hu sheep (*P* < 0.05). This indicates that CP levels during late pregnancy may influence the fat metabolism of ewes, which in turn affects the composition of MFGM proteins in colostrum.

Retinol-binding protein 4 (RBP4) was one of the up-regulated proteins with fold-change values of 1.83(M/L) and 2.22(H/L), while it showed no significant up-regulated in HP treatment vs. MP treatment. RBP4 is the only specific carrier protein for vitamin A (retinol) in the human blood (38, 39), and vitamin A is essential for human vision, immune functions, reproduction, the regulation of cell proliferation, and differentiation as well as embryonic development (39–41). RBP4 is expressed during the critical period of pregnancy and is one of the major proteins produced by the gestational body (gilts, Ramboulliet ewes, Hereford beef cows, and rat) that contributes to the transfer of retinol between the mother and the embryo (42, 43). The plasma retinol level in dairy cows decreases at the end of pregnancy, reaches its lowest level before and after delivery, and increases during the first week of lactation (44). Abd Eldaim et al. (45) found considerable amounts of RBP4 in Holstein cows' colostrum, but little in mature milk. Plasma retinol levels in newborn calves (Brown Swiss cows) are low at birth but increase significantly after consuming the colostrum (46). As colostrum causes morphological and functional changes in the gastrointestinal tract (GIT) of newborn calves (47), and vitamin A plays a role in maintaining calves' intestinal integrity (48, 49). The retinol in the colostrum may promote the maturation of the calves' GIT along with non-nutritional factors, such as insulin-like growth factor-1 (47). The results indicated that the upregulation of RBP4 in colostrum may be beneficial to the growth and development of lambs.

The functions that these differentially expressed MFGM proteins perform and the biological processes they participate in were analyzed using GO annotation and KEGG pathway analyses. The GO annotation results showed that lactose synthase activity, disaccharide biosynthetic process, and lactose biosynthetic process were only enriched in the DEPs identified between the HP and LP treatments, and the expression of the DEPs in the GO terms was all up-regulated. Lactose, which is exclusively found in dairy products, is a disaccharide composed of galactose and glucose (50). Dietary lactose needs to be first metabolized in the intestine to galactose and glucose, which can then be absorbed and enter into circulation (51). In the mammary glands, galactose and glucose are combined for the re-synthesis of lactose by lactose synthase (52). Because it is osmotically active, its physiological effect on the lactating breast is to induce fluid influx and increases milk volume (52). In the lactating mammary gland, 50 to 80% of galactose is synthesized *de novo* from glucose by lactose synthase which is highly expressed in the mammary gland during lactation (53). Wang et al. (54) reported that the difference in ADG of lambs (Hu sheep) was mainly determined by ewe milk production in the early feeding period. The results indicated that a 15.0% dietary CP level had a greater effect on MFGM proteins, which may have a positive effect on lactation and further improve the growth performance of the lambs.

The KEGG pathway analysis results showed that the porphyrin and chlorophyll metabolic pathways were enriched in both the M/L and H/L, and its related metabolite was protoporphyrin IX (PpIX). PpIX is an intermediate in heme biosynthesis and combines with ferrous iron to form the hemoglobin hemepseudogroupprotoheme IX. PpIX increases the activity of thrombopoietin (TPO) (55). TPO is the most critical regulator of platelet production. It can activate the JAK/STAT, Ras/MAPK, and other signaling pathways on megakaryocytes by binding to the surface c-Mplreceptor, inducing tyrosine phosphorylation of various proteins, including Jak2 and Stat5, activating megakaryocytes, and promoting their proliferation and differentiation to produce platelets (56–58). TPO not only has good effects in common thrombocytopenic diseases but also has protective and proliferative effects on neuronal cells, which can repair damaged neuronal cells and maintain neuronal population; it can also induce angiogenesis and play an important role in the differentiation of proliferation of progenitor endothelial cells (59). However, whether these proteins contribute to the porphyrin and chlorophyll metabolic pathways, or how they participate in these pathways remains to be explored.

## Conclusion

The growth performance of ewes and lambs of three different dietary CP levels was evaluated in this study. Different dietary CP levels during late pregnancy did not affect BW, DMI, ADG, and FCR of ewes. When the dietary CP level was increased to 15.0%, the birth weight of lambs was significantly higher than the other two treatments. Using iTRAQ proteomics technology combined with LC-MS/MS methods, a total of 1,529 MFGM proteins were identified and 286 DEPs were found. Functional analysis of differential proteins and GO annotation analysis revealed that a 15.0% CP level brought about a positive change in MFGM protein composition. Considering comprehensively, the optimum CP concentration of ewes and their offspring was 15.0%. In production, properly increasing the dietary protein level in the later stage of pregnancy is beneficial to ensure the growth and development of Hu lambs.

## Data availability statement

The mass spectrometry proteomics data have been deposited to the ProteomeXchange Consortium (http://proteomecentral.proteomexchange.org) via the iProX partner repository with the dataset identifier PXD037465.

## Ethics statement

The animal study was reviewed and approved by Animal Ethical and Welfare Committee of Ruminant Research Institution (Lanzhou University).

## Author contributions

XY and FL designed and conceived the experiments. NZ contributed to the sample and data collection. ZW analyzed the data and wrote the paper. XY and ZW reviewed and edited the manuscript. All authors have read and approved the final manuscript.

## Funding

This work was supported by Gansu Province Major Science and Technology Projects (20ZD7NA004), Gansu Province Key R&D Project (20YF8NH158 and 21YF5NH213), the earmarked fund for China Agriculture Research System (CARS-38).

## Conflict of interest

The authors declare that the research was conducted in the absence of any commercial or financial relationships that could be construed as a potential conflict of interest.

## Publisher's note

All claims expressed in this article are solely those of the authors and do not necessarily represent those of their affiliated organizations, or those of the publisher, the editors and the reviewers. Any product that may be evaluated in this article, or claim that may be made by its manufacturer, is not guaranteed or endorsed by the publisher.
